# *Arabidopsis* uses two gluconeogenic gateways for organic acids to fuel seedling establishment

**DOI:** 10.1038/ncomms7659

**Published:** 2015-04-10

**Authors:** Peter J. Eastmond, Holly M. Astley, Kate Parsley, Sylvain Aubry, Ben P. Williams, Guillaume N. Menard, Christian P. Craddock, Adriano Nunes-Nesi, Alisdair R. Fernie, Julian M. Hibberd

**Affiliations:** 1 Department of Plant Biology and Crop Science, Rothamsted Research, West Common, Harpenden, Hertfordshire AL5 2JQ, UK; 2 Department of Plant Sciences, University of Cambridge, Downing Street, Cambridge CB2 3EA, UK; 3 College of Natural and Agricultural Sciences, Center for Plant Cell Biology, University of California, Riverside, California 92521, USA; 4 Max-Planck-Insitüt für Molekulare Pflanzenphysiologie, Am Mühlenberg 1, Potsdam-Golm D-14476, Germany; 5 Departmento de Biologia Vegetal, Universidade Federal de Vicosa, Vicosa, Minas Gerais 36570-000, Brazil

## Abstract

Gluconeogenesis is a fundamental metabolic process that allows organisms to make sugars from non-carbohydrate stores such as lipids and protein. In eukaryotes only one gluconeogenic route has been described from organic acid intermediates and this relies on the enzyme phospho*enol*pyruvate carboxykinase (PCK). Here we show that two routes exist in *Arabidopsis*, and that the second uses pyruvate, orthophosphate dikinase (PPDK). Gluconeogenesis is critical to fuel the transition from seed to seedling. *Arabidopsis pck1* and *ppdk* mutants are compromised in seed-storage reserve mobilization and seedling establishment. Radiolabelling studies show that PCK predominantly allows sugars to be made from dicarboxylic acids, which are products of lipid breakdown. However, PPDK also allows sugars to be made from pyruvate, which is a major product of protein breakdown. We propose that both routes have been evolutionarily conserved in plants because, while PCK expends less energy, PPDK is twice as efficient at recovering carbon from pyruvate.

## Abstract (web summary)

During seed germination plants use gluconeogenesis to mobilize noncarbohydrate energy reserves. Here Eastmond *et al*. show that plants, unlike other eukaryotes, do not solely rely on a gluconeogenic pathway via the enzyme PCK but also use a second pathway relying on PPDK.

## Graphical abstract

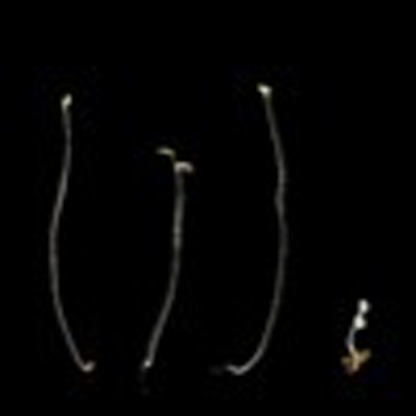

Gluconeogenesis allows seedling establishment, a critical phase in the life cycle of plants that is fuelled by the breakdown of seed reserves^1^. The efficiency of reserve remobilization has an impact on the success of plants in natural communities, but also on crops because seedling vigour is a key determinant of yield^2^. Starch, lipids and proteins can all be used, although lipid reserves are considered the most widely distributed in nature^1^. In the case of lipids, the first step in remobilization involves the hydrolysis of triacylglycerols, stored in oil bodies, to release free fatty acids and glycerol. Both products serve as gluconeogenic substrates but fatty acids account for ∼95% of the carbon. The fatty acids enter the peroxisome and are activated to fatty acyl-coenzyme A (CoA) so that they can undergo β-oxidation. The acetyl-CoA produced by this process is then converted to organic acids via the glyoxylate cycle^3^.

In plants it is widely accepted that phospho*enol*pyruvate carboxykinase (PCK, EC 4.1.1.49) then plays a pivotal role in gluconeogenesis by catalysing the conversion of the C_4_ dicarboxylic acid oxaloacetic acid to phospho*enol*pyruvate (PEP)^4^,^5^,^6^,^7^,^8^. This mechanism for PEP synthesis is generally believed to be the only physiological route by which eukaryotes bypass the practically irreversible glycolytic reaction catalysed by pyruvate kinase (EC 2.7.1.40). However, in certain bacteria an alternate pathway has also been described that allows C_4_ dicarboxylic acids to be converted via pyruvate to PEP for gluconeogenesis. For example, *Escherichia coli* can use the combined activities of malic enzyme (ME, EC 1.1.1.39) and PEP synthetase (EC 2.7.9.2)^9^,^10^,^11^,^12^, while *Acetobacter xylinum* and *Sinorhizobium meliloti* can use ME and pyruvate, orthophosphate dikinase (PPDK, EC 2.7.9.1)^13^,^14^.

PPDK is best known for its role in C_4_ photosynthesis in plants^15^. However, *PPDK* genes appear to be present in all species, regardless of their photosynthetic mechanism^16^. The possibility that pyruvate phosphorylation contributes to plant gluconeogenesis was investigated in the 1960s through the application of ^14^C-labelled pyruvate^17^ and alanine^18^ to the endosperm of germinated castor beans (*Ricinus communis*). The efficiency of sugar labelling from pyruvate was low and varied with the position of the label, consistent with pyruvate being decarboxylated rather than being converted directly to PEP^17^. A further study showed that the efficiency of sugar labelling from alanine was much greater, despite the fact that alanine is directly deaminated to pyruvate^18^. However, this discrepancy was attributed to a difference in experimental design for alanine feeding that promoted dark re-fixation of ^14^CO_2_ (ref. 18). Therefore, the data from these studies suggested that direct phosphorylation of pyruvate (for example, by PPDK) does not contribute to gluconeogenesis in castor endosperm^17^,^18^.

In the model oilseed *Arabidopsis thaliana* a series of more recent studies have shown that both *PPDK*^16^ and *PCK1* (ref. 7) are expressed following seed germination, and that disruption of *PCK1* function has a surprisingly modest effect on early seedling growth^7^,^8^. These data raise the possibility that, in spite of the existing evidence from studies on castor beans^17^,^18^, PPDK might provide an alternate route for organic acids to support gluconeogenesis in *Arabidopsis*. We therefore decided to test this hypothesis using a molecular genetic approach.

## Results

### PCK1 and cytosolic PPDK are induced following germination

**Figure 1:**
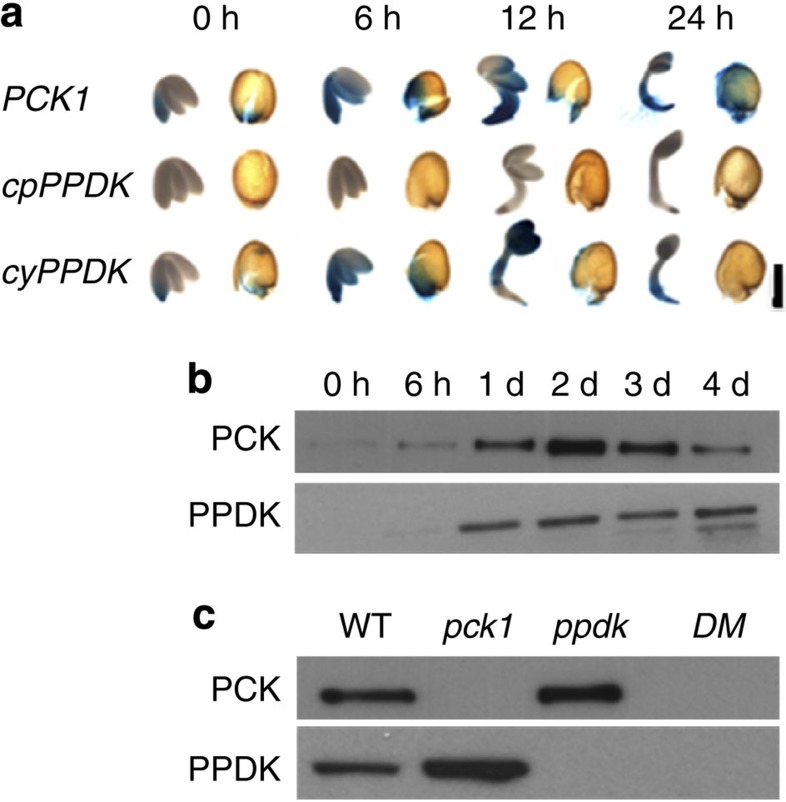
Cytosolic PPDK and PCK1 are coordinately expressed following seed germination. (**a**) Histochemical staining of transgenic plants containing genomic fusions of *PCK1::uidA* (*PCK1*), chloroplastic *PPDK::uidA* (*cpPPDK*) and cytosolic *PPDK::uidA* (*cyPPDK*) from 0 to 24 h following germination. (**b**) Immunoblots for PCK and PPDK in wild-type (WT) *Arabidopsis* seedlings from 0 to 4 days after the start of imbibition. (**c**) Immunoblots for PCK and PPDK in 2-day-old WT, *pck1, ppdk* and *ppdk-pck1* double mutant (*DM*) seedlings. Scale bar in **a**, 0.5 mm.

Analysis of transcript abundance has indicated that both *PCK1* and *PPDK* transcripts can be detected in *Arabidopsis* seeds following germination^8^,^16^. In order to compare the temporal and spatial patterns of *PCK1* and *PPDK* expression over the course of germination and early seedling growth transgenic lines expressing promoter-gene reporter constructs were created and analysed. In *Arabidopsis*, because of alternate promoter elements, one *PPDK* gene encodes both a cytosolic and chloroplastic protein^16^. Translational fusions between each gene and *uidA*^19^, coding for GUS (β-glucuronidase), show that cytosolic PPDK accumulates coordinately with PCK1 (Fig. 1a). In both cases GUS expression is first detected in the radicle of the embryo and in the micropylar region of the aleurone where the seed coat first splits and the GUS expression then spreads to the embryo cotyledons and hypocotyl as they expand (Fig. 1a). In PCK1, GUS expression also spreads across the aleurone that remains attached to the seed coat; however, in the case of cytosolic PPDK the GUS expression does not to persist (Fig. 1a). To confirm that PCK1 and PPDK proteins also accumulate in wild-type seedlings they were monitored by immunoblotting (Fig. 1b and Supplementary Fig. 1). The abundance of both proteins increases dramatically following seed germination and is highest around 2 days after the start of imbibition (Fig. 1b), when the rate of storage reserve breakdown is also known to be greatest^1^. PCK abundance then decreases as the seedling develops while PPDK remains (Fig. 1b). The expression pattern of PCK1 and PPDK in *Arabidopsis* are therefore consistent with a potential role for both proteins in gluconeogenesis.

### PCK1 and PPDK are both required for seedling establishment

**Figure 2:**
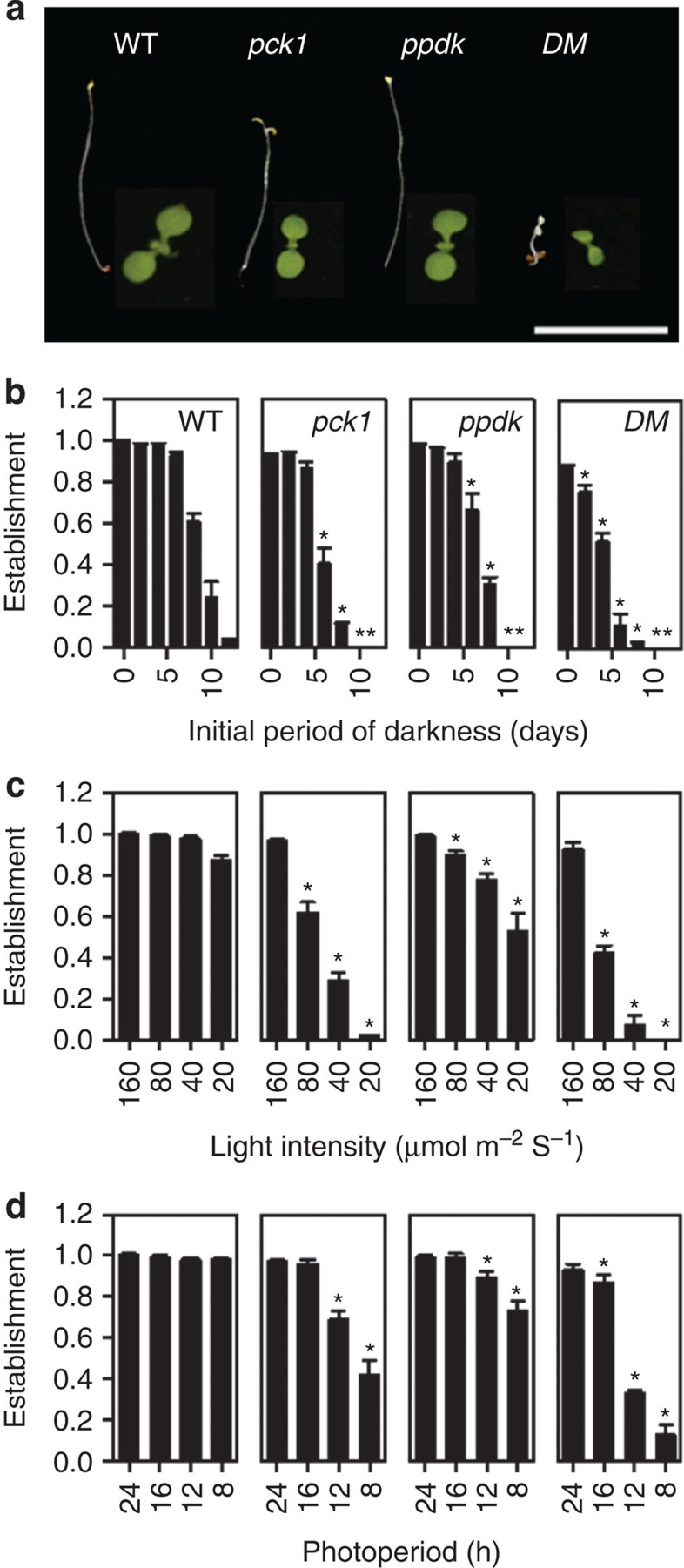
PPDK and PCK are required for normal seedling growth and establishment. (**a**) Representative images of WT, *ppdk, pck1* and *DM* seedlings grown in the dark (left) or light (right) for 5 days. Scale bar, 1 cm. The effect of (**b**) increasing periods of darkness before being transferred to light, (**c**) reduced light intensity and (**d**) reduced day length on the frequency of establishment of WT, *pck1*, *ppdk* and *DM* seedlings (as defined by the proportion of seedlings that develop true foliage leaves within 4 weeks after germination). Data are shown as means±s.e. from three batches of a hundred seeds and asterisks represent a statistical difference from WT (*P*<0.05, LSD test, *n*=3). Light intensity was 160 μmol m^−2^ s^−1^ except in **c**.

To allow us to investigate the function of PPDK in *Arabidopsis*, we obtained *ppdk* and *pck1* (ref. 8) T-DNA insertion mutants and constructed a *ppdk-pck1* double mutant. Immunoblotting confirmed that 2-day-old *ppdk* and *pck1* seedlings lack their respective PPDK and PCK proteins and are therefore null mutants (Fig. 1c). Disruption of seed reserve mobilization is known to inhibit early seedling growth and establishment in *Arabidopsis*^1^. Although PCK is considered to be essential for gluconeogenesis, *pck1* seedling growth is only modestly affected, either in the light or in darkness^7^,^8^ (Fig. 2a). Seedling development in *ppdk* appears to be normal but *ppdk-pck1* shows a severe reduction in growth rate, measured by cotyledon expansion in the light or hypocotyl extension in the dark (Fig. 2a). Early seedling growth can therefore be supported by PPDK, in the absence of PCK. The provision of mineral nutrition (Murashige and Skoog basal salts) makes no difference to hypocotyl extension in the dark, but exogenous sugar can completely rescue *ppdk*-*pck1* growth (Supplementary Fig. 2a), which is consistent with the genes functioning in gluconeogenesis^1^. The *ppdk*-*pck1* mutant could also be partially complemented using a genomic clone encoding cytosolic PPDK, while chloroplastic PPDK had no effect (Supplementary Fig. 2b). High activities of PPDK and PCK have been reported in cells around veins of *Arabidopsis*^20^. A strategy that combined use of hairpin RNA interference constructs for each gene with a vein-specific enhancer trap^21^ suggested that PPDK and PCK in cells around veins are not required for seedling growth (Supplementary Fig. 3).

Although *ppdk* seedlings appear to grow normally under optimal laboratory conditions (Fig. 2a) such conditions are uncommon in natural environments. We therefore assessed the absolute fitness of *ppdk* plants by measuring the frequency of seedling establishment (equivalent to survival) when seeds are sown under a range of suboptimal light environments that are more prevalent in nature^22^. Using this assay, fewer *ppdk* seedlings survived (*P*<0.05, least significant difference (LSD) test, *n*=3) than wild type (Fig. 2b–d). This included when access to light was delayed for a period after the completion of germination (Fig. 2b), as would be the case beneath the soil, or when light levels were low, or days were short (Fig. 2c,d). Survival of *pck1* seedlings was also reduced under suboptimal conditions and this was accentuated in *ppdk-pck1* (Fig. 2). These data suggest that both proteins are required to maximize the frequency of seedling establishment under suboptimal growth conditions. Even under optimal conditions *pck1* and *ppdk-pck1* seedlings that do establish exhibit delayed rosette growth and flowering (Supplementary Fig. 4).

### PCK1 and PPDK are both required to mobilize storage reserves

**Figure 3:**
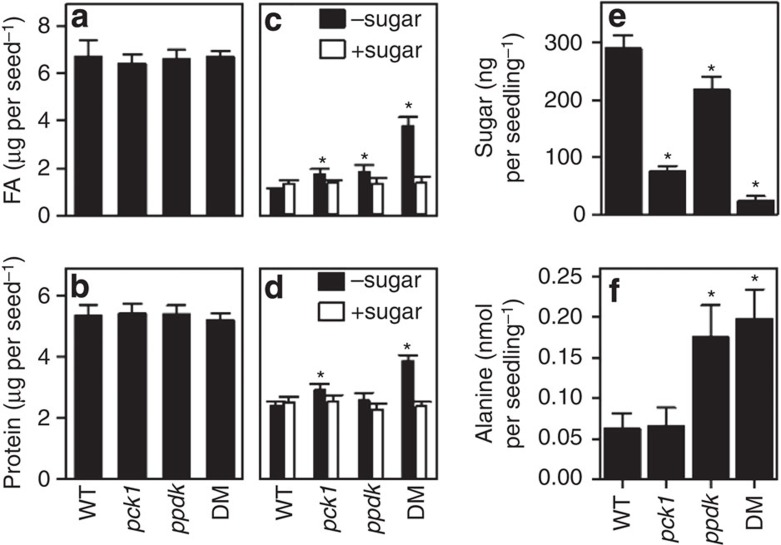
PPDK and PCK1 are required for sugar accumulation following seed germination. Quantification of total fatty acid (**a**,**c**) and protein (**b**,**d**) content in seeds (**a**,**b**) and 5-day-old seedlings (**c**,**d**) of WT, *ppdk*, *pck1* and *ppdk-pck1* grown on MS basic media, plus or minus 30 mM glucose. (**e**) Soluble sugar content in 2-day-old WT *pck1, ppdk* and *DM* seedlings. (**f**) Alanine content of two day old seedlings of WT, *ppdk*, *pck1* and *ppdk-pck1* grown on Murashige and Skoog (MS) basic media. Data in **e** are the sum of sucrose, glucose and fructose. All values are the mean±s.e. of measurements on four separate pools of seeds or seedlings and asterisks represent a statistical difference from WT (*P*<0.05, LSD test, *n*=4).

To investigate why PCK and PPDK are both needed for optimal seedling establishment, we carried out metabolite analysis. The total fatty acid and protein content of *pck1*, *ppdk* and *ppdk-pck1* seeds (Fig. 3a,b) was not significantly different from wild type (*P*>0.05, F-test, *n*=4), indicating that compromised seedling growth and establishment (Fig. 2) cannot be explained by a lack of seed reserves. Although the degradation of fatty acids and protein was impaired in *ppdk-pck1* seedlings grown in the absence of exogenous sugar, when sugar was provided the reserves were readily broken down (Fig. 3c,d). This is consistent with previous reports showing that mutant seedlings with gluconeogenic defects can still use lipids as a respiratory carbon source, particularly when sugar is provided^7^,^22^,^23^.

In wild-type *Arabidopsis* seedlings soluble sugars accumulate shortly after germination as a result of gluconeogenesis from seed-storage lipids and this is impaired in mutants that are deficient in the pathway^23^. We found that sugar levels are reduced by ∼70% in 2-day-old *pck1* seedlings, consistent with the role of PCK in gluconeogenesis^8^ (Fig. 3e). However, the *ppdk* mutant also has ∼30% less sugar, and *ppdk-pck1* has ∼90% less sugar than wild type (*P*<0.05, LSD test, *n*=4). The significant impact of *pck1* and *ppdk* on seedling sugar content, and the additive effect observed in *ppdk-pck1*, suggest that PCK and PPDK are both required for normal gluconeogenic flux.

In addition to lipids, proteins can also serve as a gluconeogenic substrate^18^, and in some seeds they are the predominant reserve^24^. The carbon backbones of alanine, cysteine, glycine, serine, threonine and tryptophan can all be metabolized to pyruvate, the substrate of PPDK, and these amino acids make up around one-third of seed-storage protein in *Arabidopsis*. Uniquely, alanine is directly and reversibly converted to pyruvate and analysis of alanine content showed that in *ppdk* and *ppdk-pck1* seedlings, it is increased (*P*<0.05, LSD test, *n*=4) approximately threefold compared with *pck1* or wild type (Fig. 3f). This suggests an important gluconeogenic function for PPDK in remobilizing carbon skeletons present in amino acids that are catabolized to pyruvate.

### PCK1 and PPDK play distinct roles in mobilization of stores

**Figure 4:**
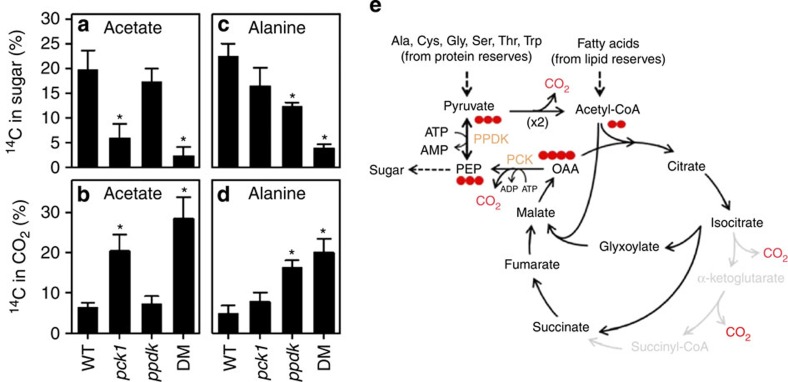
PPDK and PCK define two separate gateways for gluconeogenesis that enable efficient utilization of both lipid and protein reserves. Redistribution of ^14^C label from [2-^14^C]acetate (**a**,**b**) and [U-^14^C]alanine (**c**,**d**) into sugars and CO_2_ in 2-day-old seedlings. Data are means±s.e. from four separate pools of 100 seedlings and asterisks represent a statistical difference from WT (*P*<0.05, LSD test, *n*=4). (**e**) Schematic outlining PPDK- and PCK-dependent gluconeogenesis from lipids and amino acids during early seedling growth. Note that glycerol is also a minor product of lipid breakdown (∼5% of the carbon in storage oil) and enters gluconeogenesis downstream of PEP. A minimum of two moles of pyruvate are required to form one mole of PEP via pyruvate dehydrogenase (PDH) and the glyoxylate or tricarboxylic acid cycle, while the molar ratio is 1:1 using PPDK. However, when employing PPDK an additional ATP is necessary to regenerate ADP from AMP.

To follow the metabolism of gluconeogenic substrates derived from lipids and protein, short-term radiolabelling experiments were performed on 2-day-old seedlings using [2-^14^C]-acetate and [U-^14^C]-alanine^18^,^23^. Seedlings were labelled for 4 h and the redistribution of ^14^C into metabolic fractions was determined and expressed as a percentage of the total metabolized (Fig. 4 and Supplementary Table 1). When wild-type seedlings were fed either acetate or alanine, ∼20% of the total ^14^C was detected in sugars (Fig. 4a), suggesting that both metabolites act as gluconeogenic substrates^18^,^23^. Feeding ^14^C-acetate to *pck1* seedlings resulted in a substantial reduction in the percentage of ^14^C found in sugars (*P*<0.05, LSD test, *n*=4) but no change in the percentage of ^14^C in this fraction was observed in *ppdk* (Fig. 4a,b). On the other hand, when ^14^C-alanine was fed to *ppdk* seedlings, the percentage of ^14^C in sugars was significantly reduced (*P*<0.05, LSD test, *n*=4), while no change was seen in *pck1* (Fig. 4c,d). In *ppdk-pck1* the percentage of label found in sugars following ^14^C-alanine feeding was significantly (*P*<0.05, LSD test, *n*=4) lower than in the *ppdk* single mutant; however, the percentage of label in sugar from ^14^C-acetate feeding was similar to *pck1*. These labelling patterns are consistent with a model (Fig. 4e) in which PPDK participates in gluconeogenesis by remobilizing amino acids such as alanine that give rise to pyruvate, while PCK is primarily involved in remobilization of acetyl-CoA derived from lipids.

In *ppdk*, more than 10% of ^14^C-alanine is still metabolized to sugars versus ∼20% in wild type (Fig. 4c). The most likely explanation for this is that in the absence of PPDK, pyruvate is converted to acetyl-CoA by pyruvate dehydrogenase (EC 4.1.1.1), providing carbon skeletons to the glyoxylate or tricarboxylic acid cycles (Fig. 4e). The observation that total protein breakdown is not significantly impaired in *ppdk* seedlings (Fig. 3d) is also consistent with a diversion of flux rather than a blockage. The glyoxylate cycle in plants spans multiple subcellular compartments^1^ and thus the model in Fig. 4e is a simplification. Nevertheless, the route involving pyruvate dehydrogenase leads to the net loss of at least half the carbon as CO_2_, while conversion of pyruvate to sugar via PPDK results in no loss (Fig. 4e). Indeed, the percentage of ^14^C released as CO_2_ when *ppdk* is fed alanine is substantially greater than in wild type (Fig. 4d). Interestingly, animals and yeast lack PPDK but, by using a single pathway that combines pyruvate carboxylase (PC, EC 6.4.1.1) with PCK, also achieve no net loss of carbon as CO_2_ when pyruvate supports gluconeogenesis (Supplementary Fig. 5). When lipids are used as a gluconeogenic substrate in *Arabidopsis* the carbon conversion efficiency would be the same whether PCK or PPDK are employed because PPDK must be coupled with the decarboxylating activity of ME (Supplementary Fig. 4), but PPDK has a higher cost in ‘energy' because ATP is hydrolyzed to AMP, not ADP, and therefore requires an additional ATP to regenerate ADP via adenylate kinase (Fig. 4e). The strong impact of *pck1* on sugar labelling from ^14^C-acetate (Fig. 4a) does not support a substantial role for ME in mediating flux from lipids to PEP via PPDK. We therefore propose that plants use a PPDK-dependent gluconeogenic pathway because it allows the most efficient recovery of carbon skeletons from amino acids in seed-storage proteins that are metabolized via pyruvate, while a PCK-dependent pathway is also present because it is more ‘energy-efficient' for substrates metabolized to C_4_ dicarboxylic acids, such as lipids and other amino acids.

### PCK1 and PPDK deficiency leads to sugar-starvation responses

Compromised gluconeogenesis during early seedling growth would eventually be expected to lead to broad alterations to primary metabolism, and to induce sugar-starvation responses^23^. To address this we undertook global analysis of gene expression using the GeneChip *Arabidopsis* ATH1 Genome Array from Affymetrix^23^. Analysis of transcript abundance in 2-day-old seedlings of the mutant genotypes provided no evidence for the suppression of other genes known to play a direct role in lipid remobilization^1^. In fact, there was evidence of modest upregulation of several genes in *pck1* and *ppdk-pck1* (Table 1). However, the most striking effect on global gene expression in several of the mutants was associated with known sugar starvation marker (SSM) genes. Baena-Gonzalez *et al*.^25^ previously identified a core set of 278 such genes in *Arabidopsis*. When this list was cross-referenced with those genes more than twofold up- or downregulated in the mutants (Table 2), the *ppdk* mutant had the lowest degree of overlap (four genes), while *ppdk-pck1* had the greatest (108 genes; Table 2). A further comparison with published microarray data^23^ for 2-day-old seedlings of the glyoxylate cycle mutants, *isocitrate lyase* (*icl*) and *malate synthase* (*mls*), revealed a similar degree of overlap (180 and 64 genes, respectively) with the 278 SSM genes. Finally, *ppdk-pck1* also shares a total of 823 more than twofold up- or downregulated genes in common with *icl* and 376 genes in common with *mls* (Table 2). We conclude that when PPDK is lacking, gluconeogenesis is reduced, but that this is not sufficient to lead to a significant sugar-starvation response under optimal growth conditions. However, when both PCK and PPDK are inactive, the impact on gluconeogenesis is eventually sufficient to induce a wide-scale starvation response as has been observed in glyoxylate cycle mutants^23^.

## Discussion

Our combined data show that, *Arabidopsis*, in common with some bacteria, uses PPDK as a gluconeogenic enzyme. To our knowledge, this is the first time that two distinct routes for the entry of organic acids into gluconeogenesis have been described in a eukaryote. Uniquely, we also provide evidence that the two routes have specialized functions in lipid and protein mobilization, respectively. Our findings are also surprising because they appear to be at odds with previous radiolabelling studies using endosperm of germinated castor beans, which suggested that very little gluconeogenic flux occurs through direct phosphorylation of pyruvate^17^,^18^. RNA sequencing data have shown that *PPDK* is expressed in germinated castor beans^26^. However, there might still be variation in PPDK activity between the two species and/or tissues. Castor seeds are anatomically very different from *Arabidopsis*, storing the bulk of their reserves in their large endosperm. In contrast, *Arabidopsis* seeds possess a greatly reduced endosperm consisting of a single-cell layer (aleurone) with the majority of the reserves stored in the embryo^8^. Using a translational fusion between cytosolic *PPDK* and *GUS*^19^, we observed that expression of *PPDK* in the aleurone of germinated *Arabidopsis* seed is much less widespread and persistent than in the embryo (Fig. 1a and Supplementary Fig. 6). Therefore, variation in *PPDK* expression does exist between different storage tissues in the same seed.

It is also conceivable that PPDK does support gluconeogenesis in castor endosperm despite the inefficiency with which it metabolizes exogenous pyruvate to sugars^17^. There is now considerable evidence that metabolic channelling takes place within glycolysis in *Arabidopsis*^27^ and in castor endosperm analysis of changes in metabolic intermediates in response to anoxia has also suggested that gluconeogenesis and glycolysis occur in separate intracellular regions^28^. It is therefore possible that supplying pyruvate to endosperm slices^17^ results in this metabolite entering the mitochondrion and being respired, whereas pyruvate that is produced endogenously from amino acids, such as alanine^18^, may be channelled into gluconeogenesis. In the future, it would be interesting to investigate whether the compartmentalization and enzymatic properties of alanine aminotransferases (EC 2.6.1.2) could account for the comparatively efficient metabolism of alanine to sugars^18^. *Arabidopsis* contains four alanine aminotransferase genes and at least two are expressed in seedlings^29^,^30^.

In theory, the use of PPDK as a gluconeogenic enzyme is advantageous because it allows the most efficient remobilization of carbon skeletons from substrate that must be metabolized via pyruvate, such as certain gluconeogenic amino acids. Indeed, this subset of amino acids makes up around one-third of *Arabidopsis* seed-storage protein. It is plausible that the greater efficiency with which pyruvate can be used for gluconeogenesis is also a reason why PPDK or PEP synthase are used for gluconeogenesis in some bacteria, while animals and yeast employ an alternative route combining PC and PCK (Supplementary Fig. 5). To our knowledge there is no firm molecular evidence that PEP synthase or PC exist in plants. *Arabidopsis* does have six genes encoding ME^31^,^32^ that could allow PPDK to make a contribution to gluconeogenic flux from lipids, as has been shown for acetate in bacteria^13^,^14^. However, ^14^C-acetate labelling studies (Fig. 4a) and mutant analysis (Supplementary Table 2) suggest that this is not a major metabolic route in *Arabidopsis*.

In order for *Arabidopsis* PPDK to function in gluconeogenesis it must catalyse the synthesis of PEP *in vivo*. A recent study has shown that at cytosolic pH the specific activity of recombinant *Arabidopsis* PPDK is approximately eightfold lower in the PEP-forming direction than it is in the pyruvate-forming direction^33^. However, the *K*_m_ for pyruvate (17 μM) is ∼17-fold lower than it is for PEP and, at physiological pyruvate concentrations (∼0.1 mM), PEP formation may therefore be favoured^33^. Furthermore, although high cytosolic concentrations of pyrophosphate (PPi) should inhibit PEP formation, PPi has relatively little effect on *Arabidopsis* PPDK activity *in vitro*^33^. *Arabidopsis* has also been found to contain an H^+^-pyrophosphatase that supresses PPi accumulation during early post-germinative growth and promotes gluconeogenesis^34^.

Given our discovery that PPDK plays a role in gluconeogenesis in *Arabidopsis*, it will be important to understand how this enzyme is regulated during the phase of seed-storage reserve mobilization. Gibberellins (GA) and abscisic acid (ABA) are known to play a key antagonistic role in seed dormancy and the control of germination^35^ and there is also evidence that they regulate storage-reserve breakdown, although their role appears to vary with species and tissue^1^. In *Arabidopsis*, transcriptional induction of *PCK1* is repressed by ABA in the embryo but not the endosperm (aleurone) tissue of the seed^8^. A preliminary analysis of the effect of ABA on expression of the cytosolic PPDK–GUS fusion in embryos of imbibed seeds suggests that *PPDK* is not sensitive to transcriptional repression by ABA (Supplementary Fig. 6) and thus its regulation differs from PCK1 (ref. 8). However, PPDK may be subject to other levels of regulation. For example, it is known that the PPDK-regulatory protein (PDRP) uses phosphoryl-donor nucleotides to reversibly phosphorylate an active-site threonine residue in PPDK, thereby inactivating the enzyme and a cytosolic PDRP has been identified in *Arabidopsis*^36^, although its precise physiological role is not yet understood.

In conclusion, the progressively compromised seedling growth evident in *Arabidopsis ppdk, pck1* and *ppdk-pck1* and the reduction in carbon recovery from seed-storage reserves in both the double and single mutants clearly shows that the gluconeogenic function of both PCK and PPDK enzymes is of relevance to plant fitness. The superior carbon recovery that PPDK allows would be advantageous to both bacteria and plants when growth is dependent on substrates such as lactate and gluconeogenic amino acids that can give rise to pyruvate. *PPDK* genes appear to be present in all plant genomes that have so far been sequenced^16^ and there is also evidence that *PPDK* is expressed in the storage reserve tissues of germinated seeds from a taxonomically diverse range of species including castor bean^26^, poplar^37^ (*Populus balsamifera*), rice^38^ (*Oryza sativa*), barley^39^ (*Hordeum vulgare*) and maize^40^ (*Zea mays*; Supplementary Table 3). Although it is possible that *Arabidopsis* independently and convergently recruited PPDK for gluconeogenesis, it is therefore more likely that both bacterial and plant lineages have maintained PPDK for this purpose since their divergence around 1.3 billion years ago.

## Methods

### Plant material and growth conditions

The *PPDK* and *PCK1* insertional mutants in *Arabidopsis* ecotype Col0 (*ppdk*: SALK_073368 and *pck1–2*; SALK_032133) were identified in the SALK T-DNA collection^41^ and obtained from the European *Arabidopsis* Stock Centre (uNASC, University of Nottingham, UK). To assess seedling phenotype, metabolite content, protein and transcript levels, and to perform radiolabelling experiments, seeds were sterilized and sown on plates containing 0.8% w/v agar. Where stated, Murashige and Skoog basal salts (pH 5.7), 30 mM glucose and 20 μM ABA were also included. The seeds were then stratified for 3 days at 4 °C, subjected to a 30-min light pulse of 200 μmol m^−2 ^s^−1^ and routinely maintained in the light or dark at 22 °C for up to 5 days. Experiments to determine the frequency of seedling establishment under different light regimes were performed as described previously^22^. In soil, *Arabidopsis* seed was sown on 3:1 compost:vermiculite in controlled growth chambers with 16-h photoperiod, at 20 °C, 60% humidity and 150 μmol m^−2 ^s^−1^ light intensity. To assess seed production, plants were grown to maturity. Seeds were removed from siliques and then sieved through a 0.4-mm mesh. For hypocotyl extension assays and vegetative plant growth and development, seedlings were imaged using a digital camera and hypocotyl length determined using the ImageJ software. Green fluorescent protein and chlorophyll were imaged using a Confocal Laser Scanning Microscope as described previously^16^.

### Plasmid construction through to plant transformation

To generate hairpin constructs for both PCK and PPDK, sense and antisense regions of each gene were obtained by PCR using the primers listed in Supplementary Table 4. They were then fused to the *LIP1* intron 4 and placed under control of the GAL4VP16 upstream activation sequence as described previously^21^. Briefly, *Sac*I and *Kpn*I*Bgl*II sites were added to each sequence and used to clone sense fragments of each gene upstream of the *LIP1* intron^21^. *BamH*I and *Sac*I sites were used to allow antisense fragments of each gene to be cloned downstream of the *LIP1* intron^21^. The *Bgl*II and *Sac*I sites were then used to excise these fragments from the cloning vector and then place them into a binary vector containing the GAL4VP16 upstream activation sequence^21^. Using floral dipping *Agrobacterium tumefaciens* was used to insert each construct into *Arabidopsis* and transformants were selected on hygromycin^21^. Homozygous single insert lines for *PPDK* and *PCK* were crossed to obtain double knockdown lines.

### Metabolite measurements and radiolabelling experiments

Total fatty acid content and composition was determined using gas chromatography of fatty acid methyl esters (FAMEs) following direct transmethylation of tissue samples^22^. Pools of 20 seeds or seedlings were added to vials containing 10 μg of heptadecanoic acid internal standard. To each vial 500 μl of 1 N methanolic-HCl was added. The sealed vials were heated at 85 °C for 2 h and the FAMEs were partitioned by adding 200 μl of hexane and 250 μl of 0.9% (w/v) KCl. The hexane phase was analysed by gas chromatography with flame ionization detection. One-microlitre injections were made into a 10 m × 0.1 mm 0.2-μm film thickness SGE BPX70 column using a 250:1 split ratio and H as the carrier gas. The oven was run at 150 °C for 0.1 min and ramped to 220 °C at 16 °C min^−1^. FAMEs were quantified by the peak area with reference to the internal standard. Soluble sugars (sucrose, glucose and fructose) were extracted from pools of 50 seedlings by three sequential washes in 80% (v/v) ethanol at 85 °C for 1 h. The pooled washed were then dried in a vacuum evaporator and the sugars dissolved in water and quantified using a series of enzyme-coupled assays linked to NADPH production^42^. Alanine was measured in the same extract as sugars using the Alanine Assay Kit (Sigma-Aldrich, UK), following the manufacturer's protocol. ^14^C-labelling experiments were performed on 2-day-old seedlings following the method described in ref. 23. Pools of 100 seedlings of each genotype were incubated in the dark for 4 h in sealed vials containing 0.2 ml of 1 mM [2-^14^C]-acetate or [U-^14^C]-alanine (20 MBq mmol^−1^) and 50 mM Mes/KOH (pH 5.2), during which time-released ^14^CO_2_ was trapped in a separate well containing 5 N KOH. The soluble components of the seedlings were then extracted in 80% (v/v) ethanol at 80 °C followed by water at 40 °C. The total soluble extracts were combined, and the hydrophobic components were extracted with chloroform. The ethanol-soluble components were dried, resuspended in water and then further separated into neutral, basic and acidic fractions using ion exchange chromatography. The amount of ^14^C in each fraction was quantified by scintillation counting. In control experiments, >90% of the ^14^C in the neutral fraction was found to co-migrate with sucrose, glucose and fructose standards following paper chromatography.

### Immunoblots and gene expression analysis

Soluble proteins were extracted by homogenizing 2-day-old seedling material in 100 mM potassium phosphate buffer (pH 7.4). The homogenate was spun in a centrifuge, the supernatant removed and the protein content quantified using the Bradford Assay. For immunoblotting 10 μg of protein was loaded on a 10% SDS–PAGE gel and transferred to nitrocellulose. Antibody hybridizations were performed with primary antibodies to either PPDK^36^ diluted 1:10,000, or PCK^43^ diluted 1:1,000. GUS was visualized after staining and leaf-clearing as described previously^20^ Total RNA was extracted from 2-day-old seedlings using the RNeasy kit (Qiagen), according to the manufacturer's instructions. Quantitative PCR^8^ was conducted on multiple hairpin lines for *PCK*, *PPDK* and the double *PCK-PPDK* line using primers specific to each gene (see Supplementary Table 4). For microarray analysis, RNA amplification, labelling, fragmentation and hybridization to ATH1 genome arrays were carried out at NASC (http://affymetrix.Arabidopsis.info/). Data were pre-processed with the GeneChip robust multiarray average image-processing normalization method^44^.

### Statistical analysis

Analysis of variance was used to assess the differences in growth and metabolism between genotypes or treatments. Following significant (*P*<0.05) F-test results, the means of interest were compared using the appropriate LSD value at the 5% (*P*=0.05) level of significance, on the corresponding degrees of freedom. The GenStat (2011, 14th edition; VSN International) statistical system was used for these analyses. The significance analysis of microarray procedure^45^ was used to define significantly up- and downregulated genes (*P*<0.05) with at least a twofold increase in expression.

## Author contributions

P.J.E., H.M.A., K.P., S.A., B.P.W., G.N.M., C.P.C. and A.N.-N. performed the research. P.J.E., A.R.F. and J.M.H. designed the programme and wrote the manuscript. J.M.H. initiated and oversaw the work.

## Additional information

**Accession codes.** Microarray data are available at uNASC (http://Arabidopsis.info/) under the registration number: NASCARRAY534, and also the iPlant collaborative at http://data.iplantcollaborative.org/quickshare/d884b0174e5c7974/Exp534.zip.

**How to cite this article:** Eastmond, P. J. *et al*. *Arabidopsis* uses two gluconeogenic gateways for organic acids to fuel seedling establishment. *Nat. Commun*. 6:6659 doi: 10.1038/ncomms7659 (2015).

## Supplementary Material

Supplementary Information Supplementary Figures 1-6, Supplementary Tables 1-4

## Figures and Tables

**Table 1:** Effects of *ppdk*, *pck1* and *ppdk-pck1* on expression of genes required for gluconeogenesis from storage lipids.

Name	AGI	Log2-fold change versus WT			SAM *d*-score			*P* value		
		*ppdk*	*pck1*	*DM*	*ppdk*	*pck1*	*DM*	*ppdk*	*pck1*	*DM*
*SDP1*	At5g04040	0.12	0.49*	1.06*	0.49	1.96	4.82	0.55	0.04	0.01
*GLI1*	At1g80460	−0.07	0.29	0.52*	−0.35	1.55	3.43	0.67	0.08	0.03
*SDP6*	At3g10370	−0.02	0.49*	0.15	−0.08	2.39	0.84	0.92	0.02	0.43
*PXA1*	At4g39850	0.11	0.41	0.27	0.39	1.16	1.05	0.64	0.17	0.33
*LACS6*	At3g05970	−0.12	0.69*	0.41	−0.36	1.94	1.14	0.67	0.04	0.29
*LACS7*	At5g27600	−0.33	0.66*	0.51	−0.91	2.07	1.82	0.28	0.03	0.13
*ACX1*	At4g16760	0.03	0.16	0.48	0.20	1.07	2.17	0.81	0.20	0.08
*ACX2*	At5g65110	−0.06	0.32	0.40	−0.21	1.16	1.39	0.80	0.17	0.21
*ACX3*	At1g06290	−0.19	0.45	0.70*	−0.64	1.31	2.74	0.44	0.13	0.05
*ACX4*	At3g51840	0.01	0.46*	0.85*	0.05	3.92	8.79	0.95	0.00	0.00
*MFP2*	At3g06860	−0.17	0.94*	0.97*	−0.40	2.22	2.61	0.62	0.03	0.05
*AIM*	At4g29010	−0.06	0.05	0.09	−0.26	0.19	0.54	0.76	0.82	0.61
*KAT2*	At2g33150	−0.18	0.52*	0.50*	−0.67	2.39	2.84	0.41	0.02	0.04
*PMDH1*	At2g22780	−0.27	1.14*	0.82	−0.59	2.57	1.74	0.47	0.02	0.14
*PMDH2*	At5g09660	0.12	−0.45	−0.63*	1.01	−1.58	−4.59	0.22	0.08	0.01
*CSY2*	At3g58750	0.13	0.98*	1.22*	0.82	7.45	6.83	0.32	0.00	0.00
*CSY3*	At2g42790	−0.20	0.78*	0.92*	−0.61	2.41	3.44	0.46	0.02	0.03
*SDP2*	At3g27820	−0.46	0.36	0.45	−1.45	1.12	1.58	0.09	0.18	0.17
*MLS*	At5g03860	0.00	0.70	0.70	−0.01	1.86	2.13	0.99	0.05	0.09
*ICL*	At3g21720	−0.12	0.23	0.38*	−0.64	1.27	3.07	0.44	0.14	0.04
*PCK1*	At4g37870	0.05	−4.73*	−7.52*	0.14	−13.27	−15.07	0.86	0.00	0.00
*PPDK*	At4g15530	−7.38*	1.79*	−6.71*	−17.61	4.15	−16.85	0.00	0.00	0.00

DM, double mutant; WT, wild type.

Analysis of gene expression in 2-day-old wild type, *ppdk*, *pck1* and *ppdk-pck1* seedlings for genes known to be involved in mobilization of storage lipids^1^. Three microarray experiments were performed for each genotype and the values marked with an asterisk show a statistically significant difference from WT (*P*<0.05, SAM, *n*=3), determined using the significance analysis of microarrays (SAM) procedure^45^.

**Table 2:** Overlap between differentially expressed genes in *ppdk, pck1* and *ppdk-pck1* and genes that are known to respond to sugar-starvation.

Genotypes	Number of overlapping genes
*ppdk* versus *SSM*	4 (1%)
*pck1* versus *SSM*	41 (15%)
*mls* versus *SSM*	64 (23%)
*DM* versus *SSM*	108 (38%)
*icl* versus *SSM*	180 (64%)
*ppdk* versus *mls*	5
*ppdk* versus *icl*	9
*ppdk* versus *pck1*	14
*ppdk* versus *DM*	18
*pck1* versus *mls*	168
*pck1* versus *icl*	328
*DM* versus *mls*	376
*DM* versus *icl*	823
*DM* versus *pck1*	946

DM, double mutant; SSM, Sugar starvation marker; SAM, Statistical Analysis of Microarrays.

The overlap between genes significantly (*P*<0.05, SAM, *n*=3) more than two-fold up- or downregulated in 2-day-old *ppdk*, *pck1*, *ppdk-pck1*, *mls*^23^ and *icl*^23^ seedlings versus wild type and a set of 278 sugar-starvation marker (SSM) genes^25^. Three microarray experiments were performed for each genotype.
