# Supplementary material for: Arabidopsis uses two gluconeogenic gateways for organic acids to fuel seedling establishment
Source: Nat Commun. 2015 Apr 10;6:6659. doi: 10.1038/ncomms7659 (PMC4403315; doi:10.1038/ncomms7659)
Supplement: Supplementary Information — Supplementary Figures 1-6, Supplementary Tables 1-4 [file ncomms7659-s1.pdf]

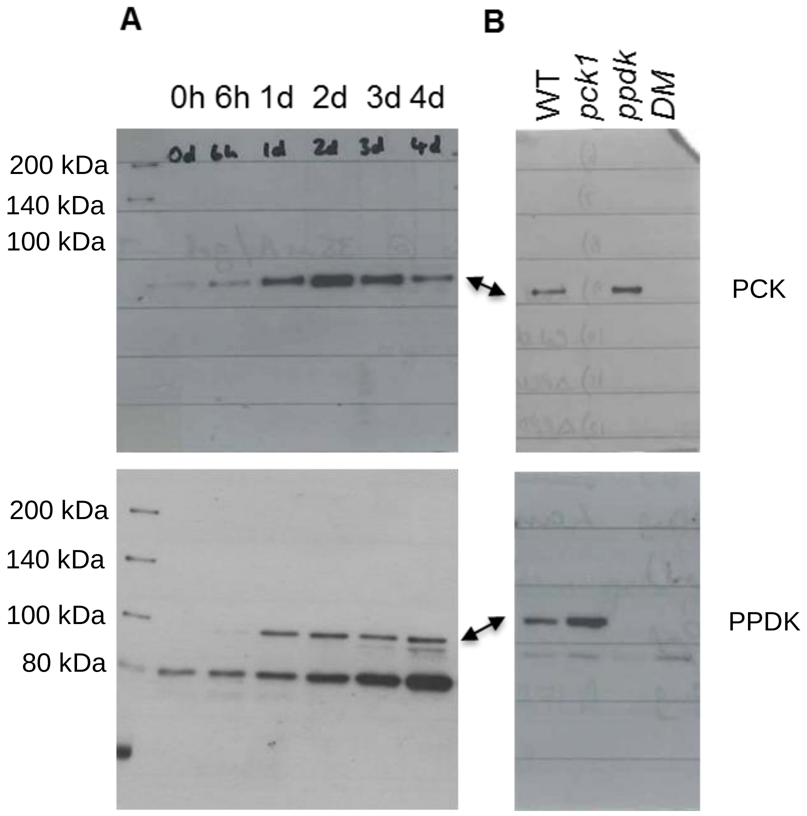

Supplementary Figure 1: Immunoblots for PCK and PPDK. In **(a)** wild type *Arabidopsis* seedlings from zero to four days after the start of imbibition and **(b)** two day old wild type (WT), *pck1*, *ppdk* and *ppdk-pck1* double mutant (*DM*) seedlings. PCK and PPDK are ~73 and ~94 kDa, respectively. Note that the lower band on PPDK blots is not specific since it is present in *ppdk* and *DM*.

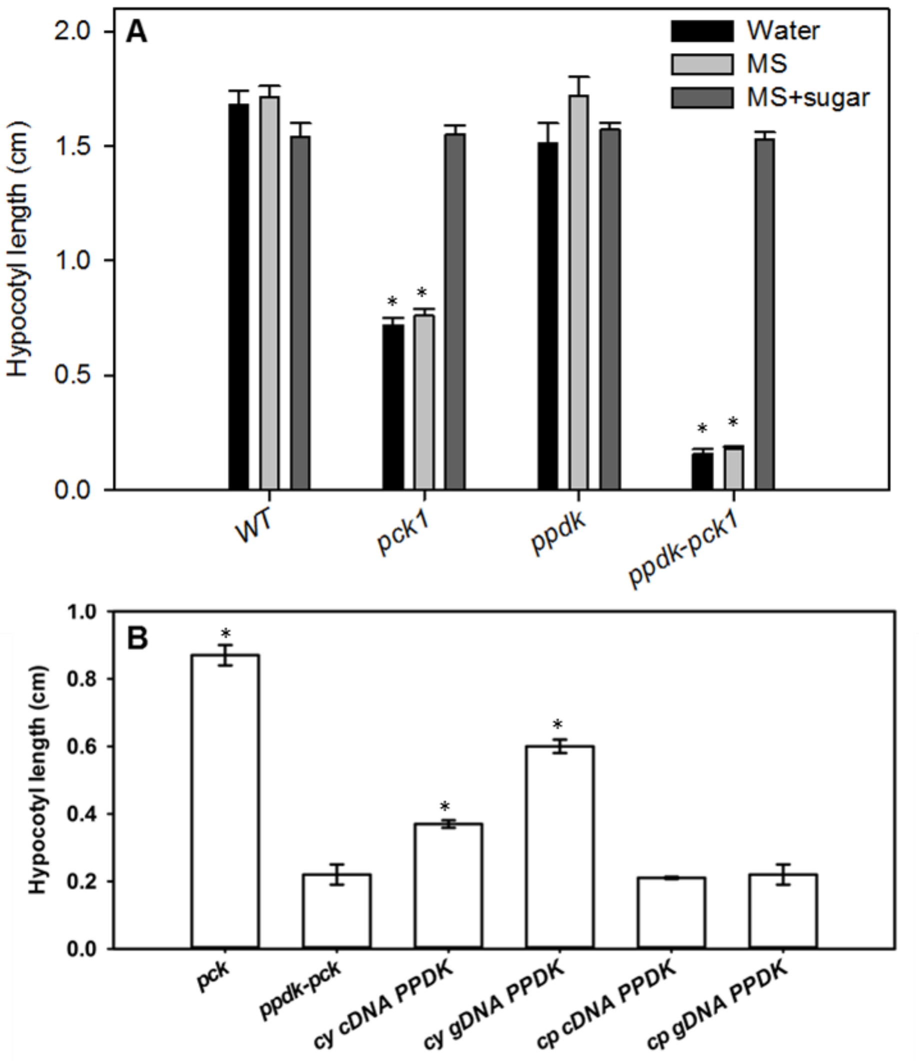

Supplementary Figure 2: Rescue of *ppdk-pck1* seedling growth. (**a**) Quantification of hypocotyl extension in seedlings of *Arabidopsis* germinated and grown in the dark for 5 days on agar plates containing either water, MS basic media, or MS plus 30 mM glucose. (**b**) Complementation of the *ppdk-pck1* double mutant with cytosolic (cy) PPDK, but not chloroplastic (cp). PPDK partially rescues the hypocotyl extension phenotype after 5 days in the dark on MS. Both cDNA and genomic (gDNA) clones were used to complement the *ppdk-pck1* mutant. Data are means ± SE of measurements on four separate batches of seedlings and asterisks represent a statistical difference ($p<0.05$, LSD-test, n=4) from WT in (a) and from *ppdk-pck1* in (b).

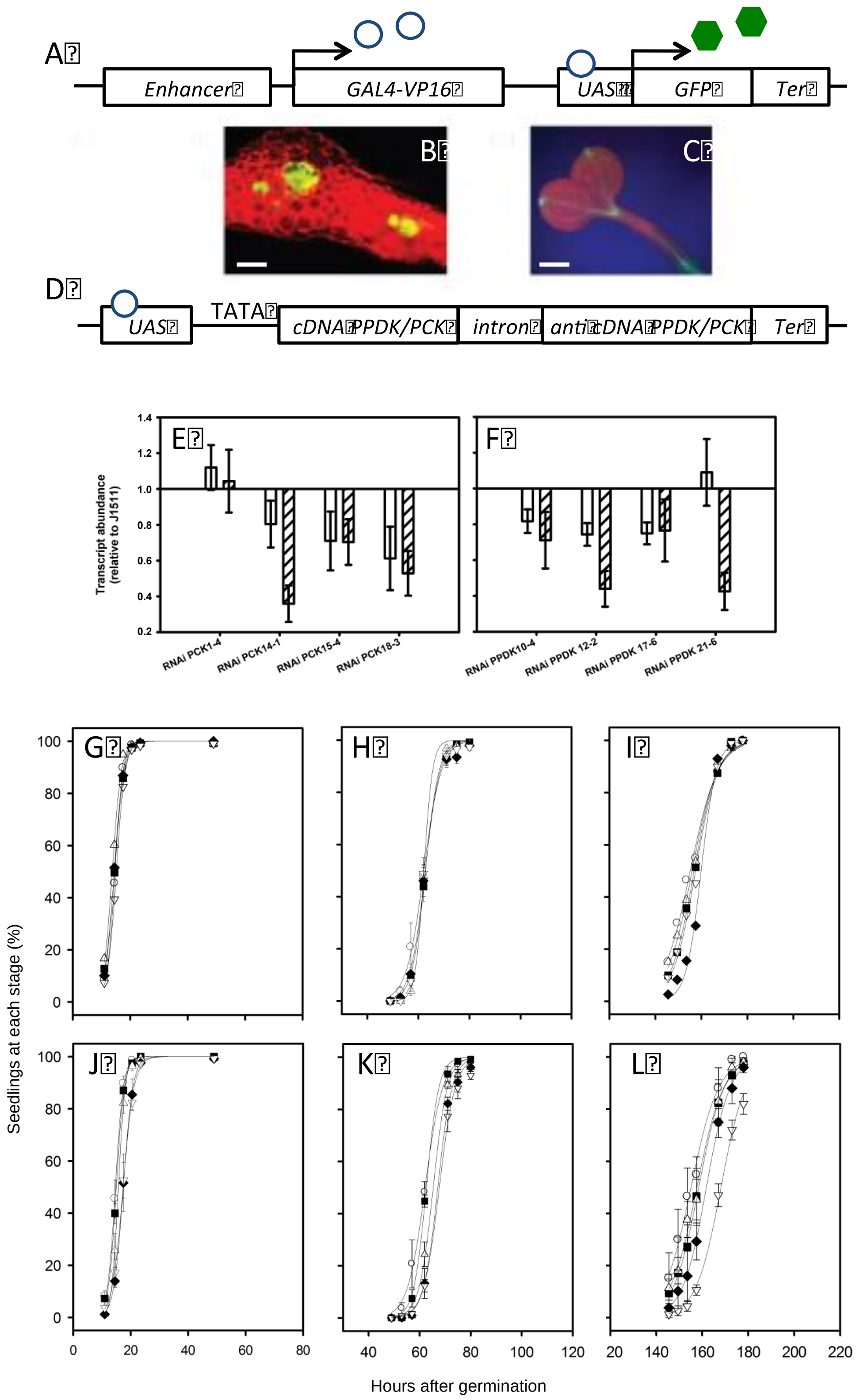
A
Enhancer
GAL4-VP16
UAS
GFP
Ter
B
C
D
UAS
TATA
cDNA PPDK/PCK
intron
anti cDNA PPDK/PCK
Ter
E
F
Transcript abundance (relative to J1511)
1.4
1.2
1.0
0.8
0.6
0.4
0.2
RNAi PCK1-4
RNAi PCK14-1
RNAi PCK15-4
RNAi PCK18-3
RNAi PPDK10-4
RNAi PPDK 12-2
RNAi PPDK 17-6
RNAi PPDK 21-6
G
H
I
J
K
L
Seedlings at each stage (%)
0
20
40
60
80
100
0
20
40
60
80
40
60
80
100
120
20
140
160
180
200
220
Hours after germination

Supplementary Figure 3: Reducing PPDK and PCK in veinal cells with RNAi activated enhancer trapping. (**a**) Scheme of GAL4-VP16 based enhancer trap construct used to *trans*-activate RNAi constructs to *PPDK* and *PCK1* in veinal cells of *Arabidopsis*. An endogenous enhancer element leads to expression of *GAL4-VP16* in specific cells. Expression of *GFP* under control of the GAL4-VP16 upstream activated sequence (UAS) leads to the cells being marked. Enhancer trap line J1511 has expression of *GAL4-VP16* and hence *GFP* in veinal cells of mature leaves (**b**), and also veins of young seedlings of *Arabidopsis* (**c**). Scale bars represent 20 µm (b) and 1 mm (c). Construct used to generate RNAi to *PPDK* and *PCK1* in veinal cells under control of the GAL4-VP16 UAS (**d**). Q-PCR for *PCK1* and *PPDK* mRNAs in leaves of four (open bars) and six (hatched bars) week old *A. thaliana* plants shows that RNAi led to a range in the extent to which transcript abundance was reduced. (**g-l**) The reduced level of *PPDK* and *PCK1* transcripts in veins of *Arabidopsis* had little impact on early seedling development of *Arabidopsis*. Reduced levels of *PPDK* transcripts did not greatly affect the time taken to complete radicle emergence (**g**), cotyledon expansion (**h**) and the production of two true leaves (**i**). Reduced levels of *PCK* transcripts did not greatly affect the time taken to complete radicle emergence (**j**), cotyledon expansion (**k**) and the production of two true leaves (**l**). Data are shown as means and one standard error of the mean. Open circles represent the control J1511 enhancer trap line, for (**g-i**) filled squares represent *PPDK* RNAi line 10.4, open triangles *PPDK* RNAi line 12.2, filled diamonds *PPDK* RNAi line 17.6 and upside-down triangles *PPDK* RNAi line 21.6, and for (**j-l**) filled squares represent *PCK1* RNAi line 1.4, open triangles *PCK1* line 14.1, filled diamonds *PCK1* RNAi line 15.4 and upside-down triangles PCK RNAi line 18.3. Scale bars in (**b**) and (**c**) are XX µm and XX mm, respectively.

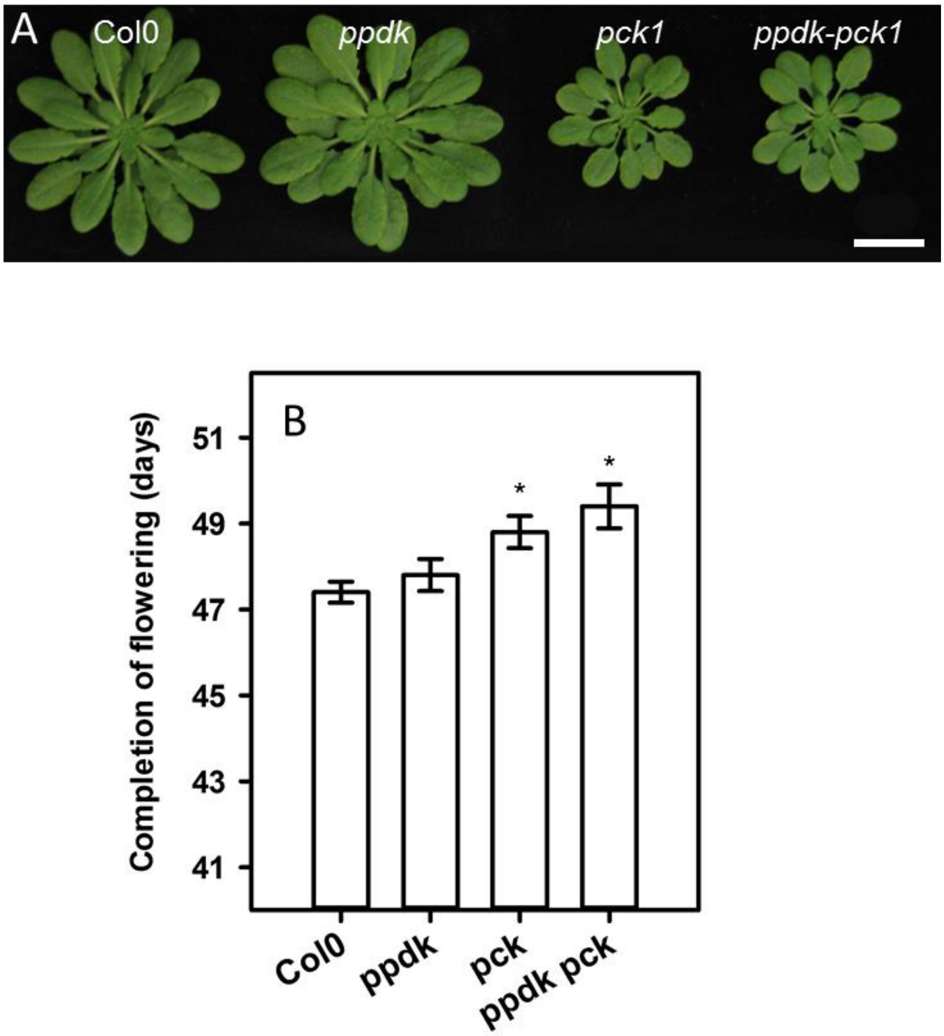

Supplementary Figure 4: Delayed growth and flowering. (**a**) Representative images of *ppdk*, *pck1* and *ppdk-pck1* genotypes sown on soil and grown for eight weeks under optimal lab conditions. (**b**) Time taken to complete flowering. Scale bar is 1 cm in (a). Data are means ± SE and asterisks represents a statistically significant difference from WT ($p<0.05$, LSD-test, n=4).

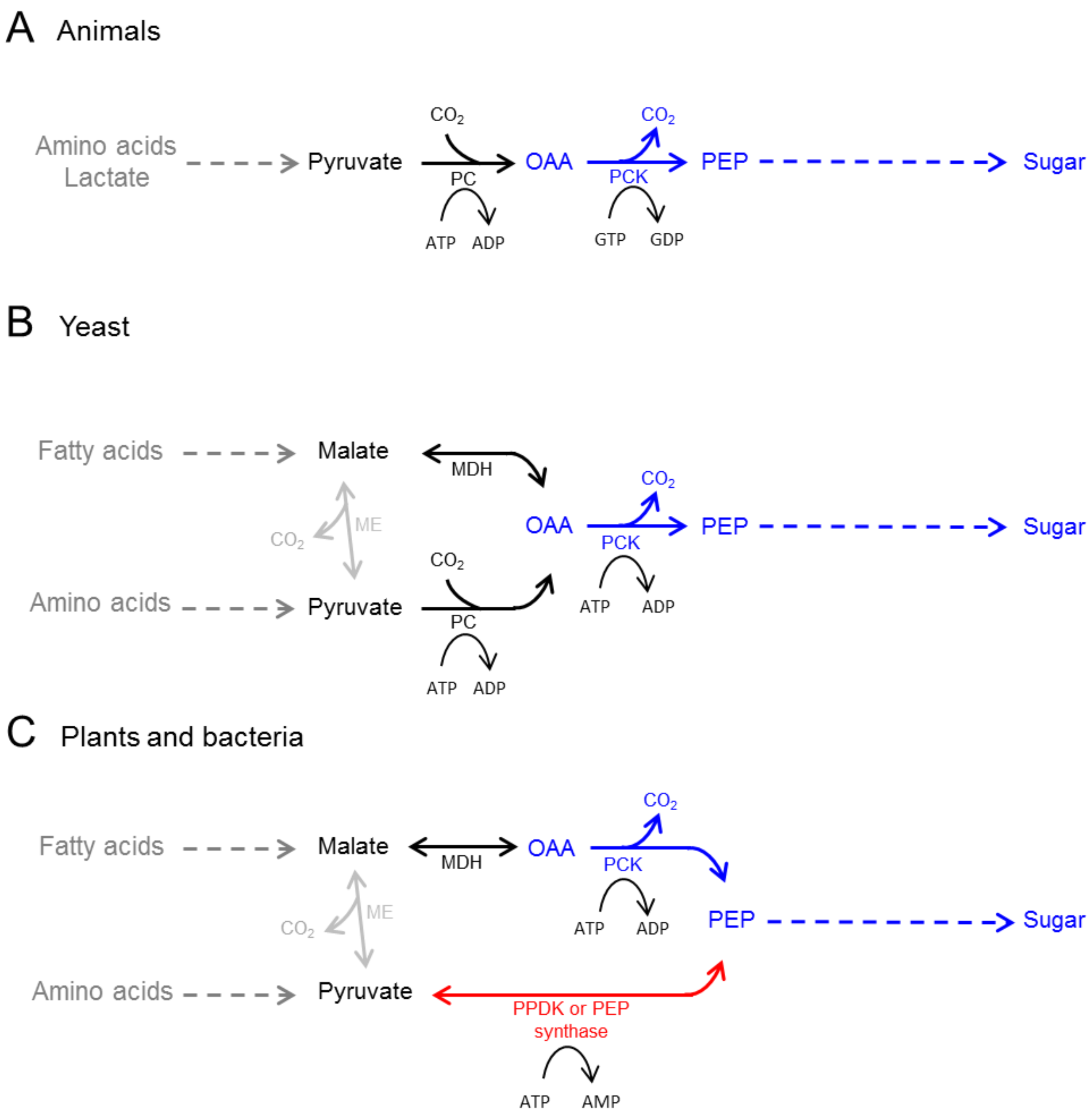

Supplementary Figure 5: Generalized schematics outlining gluconeogenesis in different organisms. (**a**) Animals (**b**) yeast and (**c**) plants and bacteria are depicted. Reactions conserved across all are annotated in blue, the PPDK reaction is in red, and multistep reactions represented by dashed lines. In bacteria, ME can allow remobilisation of carbon skeletons from fatty acids to pyruvate. PC = pyruvate carboxylase; PCK = phospho*enol*pyruvate carboxykinase; MDH = malate dehydrogenase; PPDK = pyruvate,orthophosphate dikinase; PEP synthase = phospho*enol*pyruvate synthase; ME = NAD/NADP dependent Malic Enzyme.

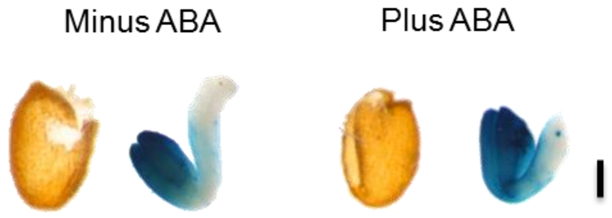

Supplementary Figure 6: Effect of exogenous abscisic acid (ABA) on PPDK expression. Representative images are shown of histochemically-stained seed coat plus aleurone and embryo from transgenic seeds containing a genomic fusion of cytosolic *PPDK::uidA*[19]. Seeds were imbibed on agar plates containing ½ strength MS basic media with or without 20 µM ABA for 2 days. Radicle emergence had occurred in the absence of ABA but not in its presence. Scale bar is 0.2 mm.

Supplementary Table 1: Redistribution of radiolabel following incubation of two day old seedlings with acetate and alanine.

Two day old dark-grown seedlings grown on ½ MS media plus 30 mM sucrose were incubated with [2-^{14}C] acetate or [U-^{14}C] alanine for 4 h. Released $^{14}CO_2$ was captured and the tissue was fractionated into ethanol-soluble material, chloroform-soluble and ethanol-insoluble material. The ethanol-soluble fraction was then sub-fractionated into neutral (sugars), basic (organic acids) and acidic (amino acids) fractions. Values are expressed as a percentage of the total and are the mean ±SE of measurements made on four separate batches of 100 seedlings. In alanine feeding experiments, the high ^{14}C content in the amino acid fraction is the result of residual substrate in the tissue[18]. The asterisks denote a statistically significant different from WT (p>0.05, LSD-test, n=4).

Species/tissue	^{14}C in each fraction (% of total)			
	CO_2	Sugars	Organic acids	Amino acids
		2-[^{14}C]Acetate		
WT	6.2 ±1.3	19.7 ±4.0	11.2 ±3.4	9.8 ±2.7
pck1	20.3 ±4.1*	5.9 ±3.0*	7.1 ±2.7*	6.0 ±2.4*
ppdk	7.1 ±2.0	17.3 ±2.7	12.9 ±3.0	10.7 ±1.9
ppdk pck1	28.4 ±5.3*	2.3 ±1.9*	6.6 ±0.8*	5.2 ±1.4*
		U-[^{14}C]Alanine		
WT	4.8 ±2.0	22.5 ±6.2	4.3 ±0.9	42.7 ±11.9
pck1	7.7 ±2.4	16.5 ±3.7*	4.9 ±1.1	45.3 ±12.5
ppdk	16.2 ±1.8*	12.3 ±0.9*	7.1 ±2.0	42.4 ±9.8
ppdk pck1	19.9 ±3.5*	3.9 ±0.8*	6.5 ±2.7	49.7 ±15.1

Supplementary Table 2: Analysis of malic enzyme mutants.

Sugar and lipid content of wild type, *nadpme4*, *nadme2-nadpme4* and *nadme1-nadpme2-nadpme4* mutant seedlings. Values are the mean ±SE of measurements on four batches of 20 to 50 seedlings grown on agar plates with ½ strength MS basal media. Values for mutants are not significantly different from WT (p>0.05, F-test, n=4).

Metabolites	WT	*nadpme4*	*nadme2 nadpme4*	*nadme1 nadme2 nadpme4*
Sugars (µg $seedling^{-1}$ at day 2)	243 ±23	250 ±33	239 ±27	230 ±21
Lipids (% drop by day 5)	87.2 ±3.4	84.9 ±2.7	86.9 ±3.4	85.3 ±4.1

Supplementary Table 3: PPDK expression in species other than *Arabidopsis thaliana*.

Data was obtained from publically available RNAseq and microarrays depositions. These data show that *PPDK* transcripts are detected during early seedling growth of castor, poplar, rice, barley and maize. For castor[26] RNAseq data are provided as FPKM (Fragments Per Kilobase of exon per Million fragments mapped) and it is notable that *PPDK* transcripts are more abundant in germinated seed (114.3 FPKM) than in the other tissues sampled (developing endosperm, leaf and male flowers). For poplar[37] and maize[40] microarray data are provided as the mean ± SD of measurements on three biological replicates. For rice[38] and barley[39] no absolute quantification is available, although for barley there is relative increase in expression between 0 and 71 h after the start of seed imbibition.

Species	Ref	Tissue	Data type	Gene / probe ID	Value
Ricinus communis	26	Germinated seed (3 d)	RNAseq	29726.m003947	114.3 (FPKM)
Populus balsamifera	37	Seedling (5 d)	Microarray	Potri.010G027800	1703 ±348
Oryza sativa	38	Aleurone plus GA (4 h)	RNAseq	Os03g31750<br>Os05g33570	>100 reads<br>>100 reads
Hordeum vulgare	39	Germinated seed (from 0 to 71 h)	Microarray	EBed01_SQ003_L20_s_at	6-fold up-regulated
Zea mays	40	Germinated seed (24 h)	Microarray	GRMZM2G097457<br>GRMZM2G306345	2910 ±189<br>40887 ±4133

Supplementary Table 4: Primer sequences used to generate the hairpin constructs and also carry out Quantitative PCR for *PPDK*, and *PCK1*.

PEPCKKpnIBglII: 5’-GAG GTA CCA GAT CTT CTG GAA CGC TAT CAA GTT TGC

PEPCKSalI: 5’-GAC GTC GAC AGT GTA TGC CAG CTT GAT TCT G

PEPCKSacI: 5’-CCG AGC TCT CTG GAA CGC TAT CAA GTT TGG

PPDKSacI: 5’-GGA CTG GTG AGC TCT TCA CTA GGA ACC CTA GCA CAG G

PEPCK QPCR FOR: 5’-GAT TCT CGC TGC TGG TCC TAT CTT

PEPCK QPCR REV: 5’-CCA TAT CGC ACC ACA TTT GGA ACA

PPDK QPCR FOR: 5’-GAA GGG TCA CGT GAG CTA CAA

PPDK QPCR REV: 5’-CCT TTG GCG AGG TAA ATC GGT AGA
